# Fibroblast growth factor receptor 1 amplification in laryngeal squamous cell carcinoma

**DOI:** 10.1371/journal.pone.0186185

**Published:** 2018-01-19

**Authors:** Jesus Monico, Brandon Miller, Luminita Rezeanu, Warren May, Donna C. Sullivan

**Affiliations:** 1 Department of Pathology, University of Mississippi Medical Center, Jackson, Mississippi, United States of America; 2 Department of Otolaryngology and Communicative Sciences, University of Mississippi Medical Center, Jackson, Mississippi, United States of America; 3 Department of Biostatistics, University of Mississippi Medical Center, Jackson, Mississippi, United States of America; 4 Department of Medicine, University of Mississippi Medical Center, Jackson, Mississippi, United States of America; Queen Mary University of London, UNITED KINGDOM

## Abstract

Fibroblast growth factor receptor 1 (FGFR1) has been noted to be amplified in a variety of squamous cell carcinomas (SCCa) of the head, neck, and lung and increased copy number (CN) is a predictor of poor outcomes. FGFR1 is a therapeutic target for lung SCCa and inhibition therapy is currently in clinical trials. Absolute quantification of FGFR1 from formalin fixed paraffin embedded (FFPE) tissue of laryngeal SCCa was examined in this retrospective study. A droplet digital polymerase chain reaction (ddPCR) was used for absolute quantitation of the FGFR1 gene CN. Of the 74 samples analyzed, FGFR1 CN analysis revealed 54% of samples had CN greater than 2 copies/cell (1.8–2.2 copies/cell), and 38% had CN values greater than 3. The mean and standard deviation FGFR1 CN was 4.17 ± 1.46 CN for African American patients (n = 41) and 3.78 ±1.85 CN for Caucasian patients (n = 31). Further, 60.9% of specimens from African Americans demonstrated increased FGFR1 CN compared to 48.4% of Caucasians. Two SCCA samples from Native American demonstrated increased FGFR1 CN (4.19 and 3.01 CN). The level of FGFR1 amplification did not correlate with tumor stage, lymph node staging, or metastasis. In this population, the proportion of patient samples with an FGFR1 amplification was three times higher than in reported for SCCA of the head and neck. Further, increased FGFR1 CN was observed in two racial groups not previously reported: African Americans and Native Americans. However, FGFR1 amplification is not prognostic in laryngeal squamous cell carcinomas.

## Introduction

Recent advances have made it possible to classify tumors not solely on the morphological phenotype, but also its molecular profile. Subcategorization of tumors can now be made based on their specific genomic alterations. More accurate classification of tumors has clinical implications with the creation of rational therapies that target exact molecular alterations. These treatments that target specific molecular alterations offer additional therapies to cancer patients and often have a better toxicity profile compared to conventional chemotherapies. These targeted therapies have been noted to be as effective as conventional chemotherapies and even promote synergistic effects [1].

Fibroblast growth factor receptor 1 (FGFR1) amplification is one of the most common genetic alterations in human cancers. FGFR1 is a receptor tyrosine kinase located on chromosome 8p12. The receptor belongs to a family of fibroblast growth factor receptors with 4 different genes, FGFR1-4. FGFR1 leads to numerous downstream effects within cells including cell differentiation and proliferation after it binds to its ligand.

FGFR1 amplification has been reported in breast adenocarcinomas [2] and described as the first actionable target in lung squamous cell carcinoma (SCCa) [3]. FGFR1 increases CN has been linked to smoking in a dose-dependent manner has been shown to be a predictor of poor outcomes in surgically treated SSCa of the lung and noted to predict a better response to traditional chemotherapy [4]. The discovery of FGFR1 amplification resulted in the immediate initiation of a phase I clinical trial of small-molecule FGFR inhibition therapy for patients with stage IV SCC disease (NCT01004224). FGFR1 amplification has also been found in local and distant metastases suggesting a clonal event in tumor progression, therefore also suggesting that FGFR1 amplification occurs early in tumorigenesis and in turn, provides promise for treating patients with advanced disease [5]. This finding is plausible considering FGFR1 plays an important role in tumor-induced vascularization.

FGFR1 amplification has also been identified in SCCa of the head and neck [6]. FGFR1 amplification in head and neck SCCa is only found in HPV-negative tumors, suggesting a separate pathway of tumor progression [7]. Similar to FGFR1 amplified lung cancer, it has also been associated with worse outcomes and strong association with tobacco and alcohol use [8]. Göke et al found FGFR1 amplification in 15% of primary SCCA of the head and neck [8]. The authors reported FGFR1 amplification highest in the hypopharynx and larynx (23% (11/47) and 18% (26/147)), respectively [8]. Although often managed with surgery, many patients with advanced or metastatic disease are either not surgical candidates or require aggressive adjuvant therapy. Currently, there are no effective targeted therapies for SCCa of the head and neck, despite it being the most common cancer of the head and neck.

To our knowledge, current research has predominantly involved studies of Caucasian patients. This study focused on determining amplification of FGFR1 in a diverse patient population with laryngeal squamous cell cancer using Droplet Digital PCR (ddPCR), a more precise and sensitive digital technology. The major advantage of ddPCR is its absolute quantification.

## Methods

Ethics Institutional approval by the University of Mississippi Medical Center Institutional Review Board (IRB) was obtained for this study. The IRB waived the requirement for informed consent.

### Tissue specimens and processing

This retrospective study employed formalin fixed paraffin embedded (FFPE) tissue samples selected from the University of Mississippi Department of Pathology paraffin block archives. A total of 80 specimens from patients with laryngeal squamous cell carcinoma were identified by a computer search of the database. Data collected from retrospective chart reviews included primary tumor stage, regional lymph node assessment, and the presence of metastasis. Of the 80 cases identified, 78 FFPE samples were available in sufficient quantity for determination of FGFR1 copy number. Two 20 micron samples of tissue were obtained from each tissue embedded block and placed in microfuge tubes. Deparaffinization solution (QIAgen, Hilden, Germany) was added to sample sections and the tubes were vortexed for 10 seconds. Samples were incubated at 56°C for 3 minutes, allowed to cool to room temperature, and centrifuged at 16,000 rpm in an Eppendorf Microfuge for 3 minutes. The deparaffinization solution was removed from the cell pellet and the tubes were incubated at 37°C for 10 minutes to allow evaporation of residual liquid. After deparaffinization, DNA was isolated by an automated extraction in a QiaCube instrument using the QIAamp DNA FFPE Tissue Kit (QIAGEN, Hilden, Germany). Quantity and quality of the DNA were evaluated using a spectral photometer (NanoDrop, PEQLab, Erlangen, Germany).

### Copy number assay for FGFR1

FGFR1 copy number was determined using a duplex primer-probe assay designed for droplet digital PCR (Bio-Rad, Inc., Hercules, CA). All assays were performed in duplicate. The probe assay consisted of unlabeled PCR primers and dual-labeled fluorescent probes for a 79 nucleotide exon of FGFR1 (FAM-labeled, Unique assay ID dHsaCP1000022) and a 98 nucleotide intron of the reference housekeeping gene RPP30 (Hex labeled, Unique assay ID dHsaCP1000485). As recommended by the manufacturer, DNA (15–50 ng/reaction) was fragmented using III (New England Biolabs, Ipswich, MA) digestion and assayed. Each 20 μl PCR reaction contained ddPCR supermix for probes (Bio-Rad, catalog number 186–3023), 18 μM primers, and 5 μM probes. Droplets were generated on a Bio-Rad QX100 as specified by the manufacturer. Samples were placed in a 96 well plate and sealed with foil for thermocycling to endpoint (T1000 Thermal Cycler, Bio-Rad) using the following protocol: initial enzyme activation and denaturation at 95°C for 10 minutes, followed by 40 cycles of 94°C denaturation for 30 seconds, 60oC annealing and extension for 1 minute, and 98°C enzyme deactivation for 10 minutes. Plates were transferred to a QX100 Droplet Reader (Bio-Rad) for analysis. Data were analyzed using the CNV mode of QuantaSoft software (Version 1.3.2.0) to assess the number of droplets positive for FGFR1, the reference RPP30, both or neither. Non-template controls were included in every run.

### Statistical analysis

Descriptive statistics were generated for the variables of race, gender, disease stage, and mortality. The SPSS 19 program (IBM SPSS Statistical Package) was employed to analyze categorical variables using Pearson’s chi-square test or Fisher’s exact test, while continuous variables were analyzed using the Mann-Whitney U test.

## Results

### Study population

A total of 78 potential retrospective records were identified, of which 74 were ultimately included in the study. The study population included 31 Caucasians (42%), two Native Americans (2.7%), and 41 African Americans (55.4%; see Table 1). There were 5 females and 69 males. The proportion of African American in this study is higher than in previously published studies of FGFR1 copy number in various types of cancer [6, 8]. Tobacco use was reported for 65% of Caucasians and 54% African American.

**Table 1:** Comparison of groups with FGR ≥ 3.0 versus FGR < 3.0 for demographic and outcome measures.

	FGR<3.0 (N = 46)	FGR≥3.0 (N = 28)	All subjects (N = 74)	p-value
**Demographics**				
Race				
African American	50.0%	64.3%	55.4%	0.2305
Other	50.0%	35.7%	44.6%	
Gender				
Female	6.5%	25.0%	13.5%	0.0241
Male	93.5%	75.0%	86.5%	
**FGFR Assay**				
FGR CN Median (range)	2.09 (0.79, 2.99)	4.44 (3.00, 9.71)	2.61 (0.79, 9.71)	-NA-
**Tumor Characterization**				
T Stage	(N = 41)	(N = 27)	(N = 68)	
1,2	24.4%	25.9%	25.0%	0.8862
3,4	75.6%	74.1%	75.0%	
N Stage	(N = 42)	(N = 27)	(N = 69)	
0	73.8%	81.5%	76.8%	0.4612
>0	26.2%	18.5%	23.2%	
M Stage	(N = 39)	(N = 28)	(N = 67)	0.9999
0	97.4%	100.0%	98.5%	
>0	2.6%	0.0%	1.5%	
**Outcome**				
Status	(N = 44)	(N = 26)	(N = 72)	
Alive	64.3%	63.6%	63.9%	0.9554
Dead	35.7%	36.1%	36.1%	

Proportions are conditioned on FGR groupings. Categorical variables analyzed using Pearson’s chi-square test or Fisher’s exact test, continuous variables analyzed using Mann-Whitney U test.

### Tumor samples and FGFR1 ddPCR assay

A total of 78 FFPE samples were obtained for determination of FGFR1 copy number. Two 20 micrometer samples of tissue were obtained from each tissue embedded block. DNA was isolated from 74 samples in sufficient concentration (≥ 10 ng/μl) and condition for CN analysis. Four samples failed to yield DNA with sufficient concentrations or quality even on isolation from a second 20-micrometer sample of tissue. The relatively small amount of tissue embedded in the block was the most likely factor contributing to the inability to obtain DNA suitable for PCR in these four samples. These samples repeatedly failed to produce positive signals for either the FGFR1 or RRPO gene in the ddPCR assay. Previous studies have shown that DNA suitable for the production of amplicons between 100 and 300 bp are readily generated from FFPE samples from a variety of tissues and varying length of storage [9] and high purity DNA has been shown to be obtained employing the DNA extraction method employed here [10].

**Fig 1:**
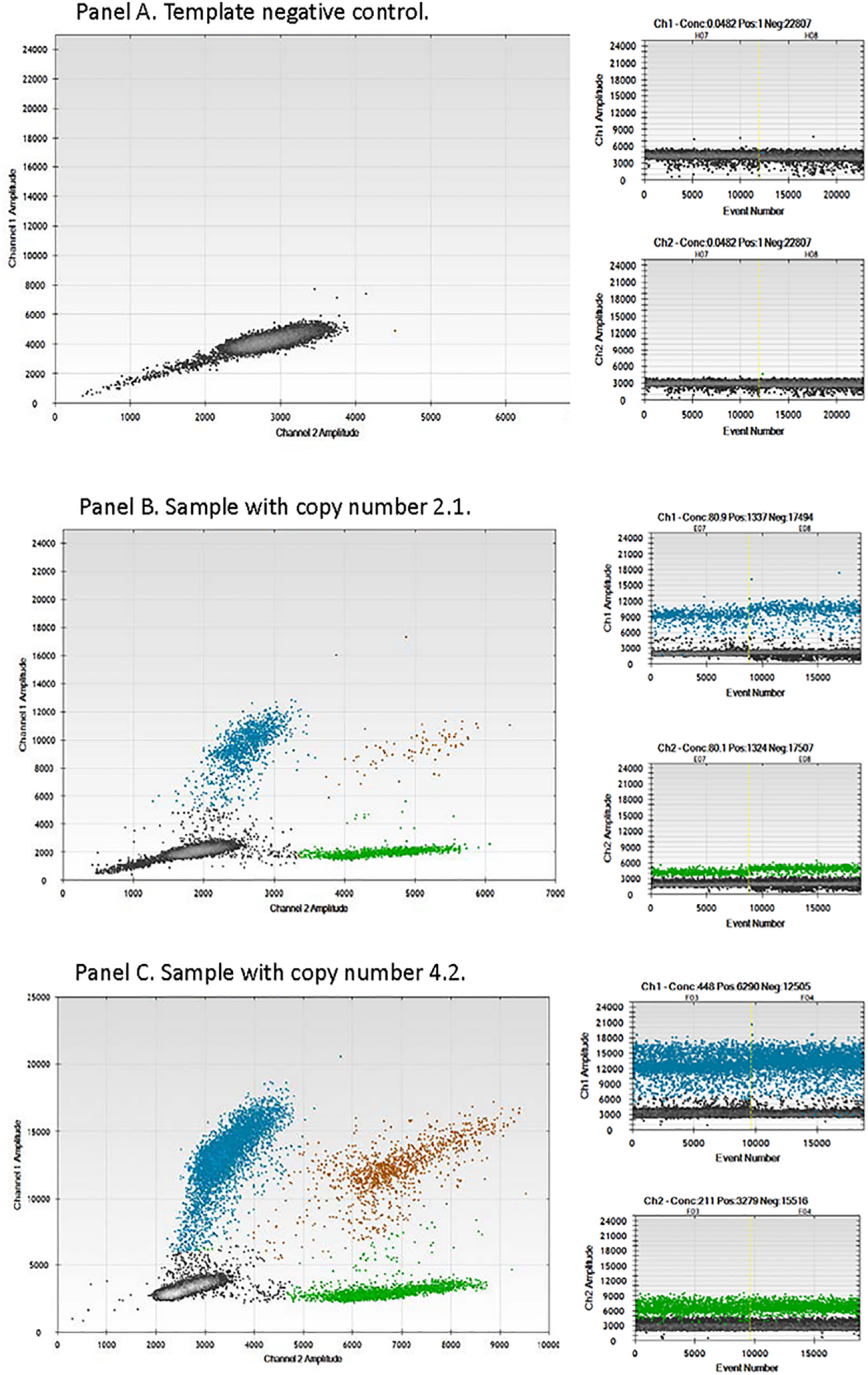
Droplet digital PCR analysis of formalin fixed paraffin embedded laryngeal squamous cell carcinoma. *FGFR1* was detected using a FAM labeled probe and compared to the HEX labeled housekeeping gene RPP30 signal. Panel A, non template control; Panel B, CNV = 2.1; Panel C, CNV = 4.2. Blue color—FGFR1 alone; Brown color—FGFR1+RPP30; Green color—RPP30 alone; Black color—below detection threshold.

Tissue samples from surgical dissections are heterogeneous by nature, containing varying mixtures both normal and tumor cells. This heterogeneous state presents a challenge in detection of gene amplification and may result in decreased sensitivity. The use of ddPCR assays has been shown to accurately detect with a high sensitivity copy number of specific genes in mixed cell samples [11]. The commercial assay employed in this study has been shown to have an assay specificity of > 95% and yield copy number calls within 10% of expected (Bio-Rad Bulletin 6444). Thus, the normal range for FGFR1 copy number employed was 1.8–2.2 copies/cell, copy number (CN). The ddPCR assay detected distinct signals for the FAM-labeled FGFR1 gene (Fig 1, Channel 1, y-axis) and the HEX labeled housekeeping gene (Channel 2, x-axis). Four distinct droplet populations were observed, corresponding to absence of signal (-/-), the presence of FGFR1/absence of RPPO30 (+/-), the presence of both signals (+/+), and the absence of FGFR1/presence of RPPO30 (-/+). Non-template controls (-/-) produced less than 3 positive signals for either labeled probe (Fig 1, panel A) while specimens with copy numbers of 2 and 4 detected positive signals in each of the other populations, +/-, +/+, and -/+, as shown in Fig 1, Panels B and C.

Of note, 3/74 samples had CN less than 2 (0.79, 1.7, and 1.2 CN). FGFR1 CN analysis revealed: 54% of samples had CN greater than the reported normal value (1.8–2.2 CN), and 38% of samples had CN values greater than 3 (range 3–9). The mean and standard deviation FGFR1 CN was 4.17 ± 1.46 (range 2.21–9.07) CN for African American patients and 3.78 ±1.85 (range 2.22–9.71) CN for Caucasian patients. Further, 60.9% of specimens from African Americans demonstrated increased FGFR1 CN compared to 48.4% of Caucasians. Both Native American patient samples demonstrated increased FGFR1 CN, with a mean and standard deviation of 3.60 ± 0.83.

### FGFR1 CN and demographics

There were n = 80 records collected over approximately a 15-year period. Of these, 6 subjects did not have a recorded FGR1 CNV so the analyses were performed for complete cases based on n = 74 individual subjects. For subsequent statistical analysis, comparisons for demographic and outcome measures were made for FGFR1 CN of <3 and ≥3. Demographically, even though there was a higher percentage of African Americans in the ≥ 3 CN group, this difference was not statistically significant (p-value = 0.2305, OR = 1.80, 95% CI [0.69, 4.73]). There was a significant difference between the males and females, with the FGFR1 CN ≥3 group having a higher proportion of females (p-value = 0.0241, OR = 4.78, 95% CI [1.12, 20.36]).

### FGFR1 CN and clinical characteristics

The American Joint Committee on Cancer (AJCC) staging criteria for cancer was employed for characterization of tumors. Tumor stage data was available for 68 of the 74 specimens evaluated. AA had more tumors in stage 4 than their Caucasian counterparts (24 vs 17), but this difference was not statistically significant (confidence interval of 92.14%). In order to determine whether copy number correlated with tumor stage, tumor stages 1–2 and 3–4 were grouped and compared to FGFR1 CN (Table 1). No significant difference between <3 and ≥3 FGFR CN was observed for tumor stage (p-value = 0.8862, OR = 1.08, 95% CI [0.35, 3.32]). No differences were noted when regional lymph node involvement (p-value = 0.4612, OR = 1.56, 95% CI [0.48, 5.13]) or metastasis staging (p-value = 0.9999, OR-zero cell, not calculated; only 1 metastasis was recorded) was considered. Further, there was no statistical difference for mortality status between the two FGFR groups (p-value = 0.9554, OR = 1.02, 95% CI [0.38, 2.76]).

### Univariate and multivariate analysis

**Fig 2:**
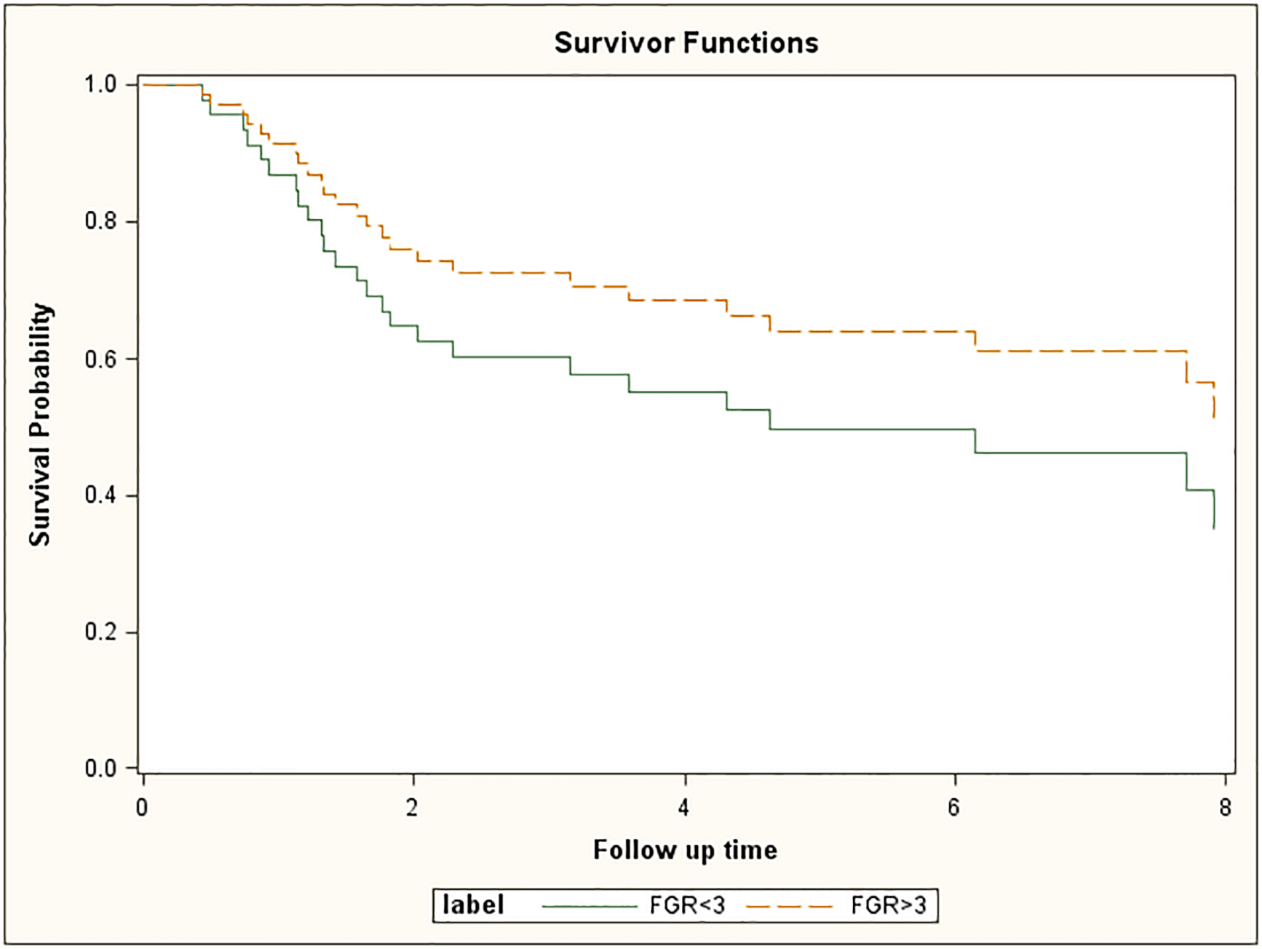
Survivorship as a function of follow up time. There is no difference in the *FGFR1* groups for status [p-value = 0.9554, OR = 1.02, 95% CI (0.38, 2.76)].

The univariate and multivariate Cox proportional hazards model were used to determine the hazard ratios (95% Confidence Interval) using patient status (alive) and the follow-up time (years) as the outcome of interest. Survival curves were calculated using the Kaplan and Meier method, and differences between curves were analyzed by the log-rank test. Results are shown in Fig 2. The factors of gender, race, FGFR1 copy number, tumor stage (T = 1,2), and lymph node stage were evaluated for 70 subjects (n = 26 dead, 44 = alive) and the results are shown in Table 2. None of the factors were significantly associated with survival. The hazard ratios for gender, race, and FGFR1 amplification were potentially clinically important individually, but not when placed in a multivariate model.

**Table 2:** Results of univariate and multivariate Cox proportional hazards model reported as hazard ratios (95% Confidence Interval) using status (alive) and follow up time (years) as the outcome of interest.

	Univariate	Multivariate
Race		
African American		
Caucasian/Native American.	1.71 (0.79, 3.73)	1.39 (0.62, 3.09)
p-value	0.1715	0.4230
Gender		
Female		
Male	1.82 (0.55, 6.07)	1.35 (0.38, 4.75)
p-value	0.3294	0.6427
*FGFR1* CNV		
FGR<3.0	1.58 (0.70, 3.52)	1.46 (0.63, 3.38)
FGR≥3.0		
p-value	0.2691	0.3742
T Stage		
1,2		
3,4	1.09 (0.37, 3.22)	
p-value	0.8758	
N Stage		
0		
>0	1.09 (0.37, 3.22)	
	0.8758	

## Discussion

### Quantitation of FGFR1

FGFR1 amplification in head and neck squamous cell carcinoma has recently been reported to be most common in the larynx[8]. We decided to measure FGFR1 amplification in a more diverse patient population. This is the first study to employ absolute quantitation of FGFR1 CN by ddPCR in laryngeal SCCa. The need to standardize the methods employed to define amplification of FGFR1 in different tumor types have been noted [12, 13]. FGFR1 fluorescent in situ hybridization (FISH) was evaluated and high-level amplification was defined as a ratio of FGFR1 to the centromere 8 (CEN8) marker of ≥2 or a defined number of tumor cells with various levels of FGFR1 [12]. Another study correlated FISH and silver in situ hybridization with morphologic, histochemical assays as well as pathologic and clinical characteristics [13]. The utility of archival FFPE tissues in nucleic acid testing has been demonstrated for a number of platforms, including ddPCR [14, 15, 16]. In a variety of tissues ddPCR can reliably detect and quantitate gene amplification. The assays can be easily set up, rapidly run, and require a minimum of turnaround time, and therefore considered an excellent screening tool for copy number variants.

### Prevalence of FGFR1 amplification

A higher percentage of patients with FGFR1 CN ≥3 were noted as compared to previous reports: 38% vs 18% in patients with laryngeal SCCa[8]. In addition, laryngeal SCCa is lower compared to esophageal SCCa which was recently found to have an amplification rate of 8.6 [17]. Previous work had not found FGFR1 amplification in SCCa of the cervix, penis, or skin, again with a suggested link to smoking [8]. Using the more sensitive ddPCR assay, the percentage of FGFR1 CN variants increased to 54%, incorporating values between 2.2–3 CN. It can be speculated that samples in this range may have a higher ratio of normal to tumor tissue in the individual samples since microdissection of specific sections of specimens from paraffin blocks was not used for extraction.

This study documented the first cases of FGFR1 amplification in African American and Native American patients. The findings are significant as previous gene amplification had been shown to vary upon ethnicity such as epidermal growth factor receptor in lung cancer patients of Asian ethnicity [18]. Identification of 3 samples with apparent deletions of FGFR1 was also observed. The complete absence of FGFR1 protein has been previously reported in a subset of oral SCCa’s. The authors hypothesized that this may be due to a loss of heterozygosity associated with later tumor progression [19]. Although the relatively small sample size in this study prohibited statistical analysis, African American patients had higher rates of increased FGFR1 CN than Caucasian patients (60.9% vs. 48.4%). Of interest, FGFR CN ≥3 occurred more frequently in females in this very small population.

### Association with tumor stage and prognostic value

A recent systematic review of FGFR family members as prognostic indicators in squamous cell carcinomas of the head and neck found 12 original articles meeting the criteria for analysis [16]. Young and colleagues (2013) found FGFR1 amplification in 10% of cases with oral tongue squamous cell carcinoma but no correlation to survival [20]. An extensive study of FGFR1 amplification in lung cancer also failed to detect a relationship between copy number and patient outcome [13, 21]. In studies of non-small cell lung cancer, FGFR1 amplification has been significantly associated with shorter overall survival and shorter disease-free survival on univariate analysis but was not statistically significant on multivariate analysis [22]. In contrast, FGFR1 amplification has been associated with parameters for worse outcomes, specifically smoking and alcohol consumption [4, 6]. Göke and colleagues later reported that FGFR1 mRNA or protein expression plays a more important role than gene copy number alone [23].

Some limitations to this study are the sample size, greater portion of males compared to females, and likely analysis of some normal tissue with tumor tissue present on our paraffin blocks. While some of our samples were primary tumors and others salvage, previous work has shown no difference in FGFR1 amplification after radiation or chemotherapy [8]. Future research will focus on obtaining larger sample sizes that will provide a more robust statistical analysis in order to compare tumor stage, tobacco/alcohol use, survival, and demographic data.

This study complements other FGFR1 studies that demonstrate genomic profiling as an approach to further understand, classify, and treat cancer of the head and neck. After more data is gathered, FGFR1 amplification may be used as a marker for aggressive disease. In addition, it may be useful to delineate whether a patient with SCCa at two different locations has 2 primary cancers or metastatic disease, again imparting important information about prognosis. Further research will need to be done to better understand the tumorigenesis of FGFR1 mediated malignancies of the head and neck and how they differ from human papillomavirus-derived disease. Laryngeal SCCa is difficult to study due to frequently small sample sizes and the fact that there is no standardized criterion for FGFR1 amplification. Hopefully, future work will contribute to the treatment options to offer patients with SCCa of the head and neck as they often have poor functional and overall outcomes despite recent improvements in adjuvant therapies.

In conclusion, a quantitative ddPCR assay was employed to detect FGFR1 gene amplification, offering a more rapid turnaround time for detection as well as the potential for highly sensitive, quantitative CNV analysis. This study documents FGFR1 amplification in African Americans and Native Americans as well as Caucasian populations. The overall incidence of amplification is three times higher in our population than previously reported populations. Further, the study documented the presence of increased CN in two racial groups not previously reported: African Americans and Native Americans. There were no statistical differences in the number of individuals with increased FGFR1 CN or in the absolute CN noted, based on race. Further, there is no correlation between FGFR1 CN and prognosis. However, it remains a potential indicator of targeted chemotherapy.

## Supporting information

S1 File FGFR1 and RPPO signals samples 1–31. (CSV)

S2 File FGFR1 and RPPO signals samples 32–57. (CSV)

S3 File FGFR1 and RPPO signals samples 58–80. (CSV)
